# A Study of the Brain Functional Network of Post-Stroke Depression in Three Different Lesion Locations

**DOI:** 10.1038/s41598-017-14675-4

**Published:** 2017-11-01

**Authors:** Yu Shi, Yanyan Zeng, Lei Wu, Ziping Liu, Shanshan Zhang, Jianming Yang, Wen Wu

**Affiliations:** 1 Department of Rehabilitation, Zhujiang Hospital, Southern Medical University, Guangzhou, 510282 China; 2 Department of Radiology, Zhujiang Hospital, Southern Medical University, Guangzhou, 510282 China

## Abstract

Research on the mechanism of post stroke depression (PSD) is the key way to improve the treatment of PSD. However, the functional brain network of PSD has not been entirely supported by the results of functional magnetic resonance imaging (fMRI) studies. The aims of this study are to investigate the brain response of PSD in three different lesions. The brain responses of the three PSD subgroups were similar. However, each subgroup had its own characteristics of the brain network. In the temporal lobe subgroup, the right thalamus had increased degree centrality (DC) values which were different from the other two subgroups. In the frontal lobe subgroup, the left dorsolateral prefrontal cortex, caudate, and postcentral gyrus had increased DC values which were different from the other two subgroups. The hemodynamic response of PSD indicates that PSD has activities of similar emotional networks, of which the negative network realizes its function through the limbic system and default mode network. The brain network has unique characteristics for different lesion locations. The neurological function of the lesion location, the compensatory mechanism of the brain, and the mechanism of integrity and locality of the brain are the important factors in the individual emotional network.

## Introduction

Post-stroke depression (PSD) is considered to be the most frequent and important neuropsychiatric consequence of a stroke that negatively affects patient outcome^1^. A recent systematic review revealed that the frequency of PSD is 33% (95% confidence interval [CI], 29% to 36%)^1^. Persistent depression not only increases disease deterioration, but also causes reduced social function, and increases the risk of suicide^2^. Consequently, research on the mechanism of PSD is vital and provides an invaluable tool to improve the treatment efficacy of PSD.

Brain imaging technology is increasingly being used by researchers to study the mechanism of PSD^3,4^. Studies have shown that several brain areas play an important role in the mechanism of PSD, such as the prefrontal cortex (PFC), amygdala (Amyg), thalamus, and hippocampus (HP)^5^. In our previous research, we found that the gray matter density (GMD) of the anterior cingulate cortex (ACC), dorsolateral prefrontal cortex (DLPFC) and HP was lower in PSD patients^6^. Based on these observations, some researchers have summarized as the mechanism of emotional circuit imbalance of PSD^7^.

However, most previous studies (including that of the authors’ group) did not include an analysis of the brain network of PSD in different patient types^8,9^. Instead, all types of patients were grouped together for analysis, without paying attention to the basic factors of stroke such as type of stroke, location and size of lesion, and duration of disease. Many studies have shown that factors such as type of stroke and duration of disease, affect the development of PSD^10^, and that there is an association between PSD and specific lesion locations or hemisphere. Mood disorders of PSD are more likely to occur at specific lesion locations of stroke, such as the ganglia and left PFC^11^, which highlights that different lesion locations have different effects on the disease. In addition, it is well known that the frontal lobe plays an important role in emotional regulation, and frontal lobe stroke can result in mood disorders. In clinical practice, however, parietal lobes which are not closely related to the emotional network also contribute to depressive symptoms, suggesting that stroke at different lesions/lobe may have unique brain network characteristics acting on the emotional network. Therefore, grouping all types of patients together for analysis would result in a high heterogeneity of subjects. As such, it is important to conduct a brain network research on single types of PSD patients, thereby improving the reliability of the conclusions.

In recent years, resting-state fMRI (rs-fMRI) has been extensively used to understand the mechanism of brain function^12^. Among the methods of rs-fMRI data analysis, functional connection (FC) and independent component analysis (ICA) are the most commonly used for examining connectivity patterns in distinct brain regions^13,14^. In the FC method, researchers generally need to select an a priori defined region of interest (ROI) before analysis. However, for poorly understood brain networks, it is very difficult to select an a *priori* defined ROI, and this affects the reliability of the conclusions. Compared with the FC method, the ICA method may be performed without a *priori* selection^15^. although it is difficult to distinguish the results as physiological noise or real brain response; this also affects the reliability of the results. At present, an increasing number of researchers are focusing on a new method termed degree centrality (DC)^16^. DC can count the number of direct connections for a given voxel in a brain network and reflect its FC within that network, without requiring an a *priori* defined ROI. Based on voxel level, DC technology takes each element as a node, and then calculates the amount of each node connecting with other nodes. This indirectly reflects the position and importance of the node or brain regions in the whole-brain networks^17,18^. Converging evidence indicates that DC is one of the main topological properties measured in graph theory analysis, and provides as an effective index. It has been widely used to identify changes in resting-state functional networks in mental illness, including depression^19^, schizophrenia^20^ and autism. However, no literature is available on PSD research based on the DC method. This method provides a promising tool for the elucidation of the neural basis of PSD because it does not require an a *priori* defined ROI.

The frontal, temporal and parietal lobes serve as the main donor brain areas of the internal carotid artery system, the three lobes are closely related to advanced brain function (i.e. emotion, cognition and memory) and is the predilection site of stroke^21^. Stroke in these brain regions is also the focus of current research.

To reduce heterogeneity, we chose patients with three lesion locations (temporal, frontal and parietal lobes) with similar stroke type, duration, size of lesion, and other basic factors. Moreover, healthy volunteers with approximate conditions were selected as the blank control group (baseline). Our aim in the present investigation was to compare the brain response of PSD and non-PSD (non-depression after stoke) subjects by grouping fMRI datasets into three different lesion locations. We hypothesized that each different type of PSD has a unique feature of the brain network, but there are also some similarities between them. Through this research, we will contribute to the further understanding of the mechanism of PSD, and provide a bridge for future studies.

## Method and Subjects

### Participants

**Figure 1:**
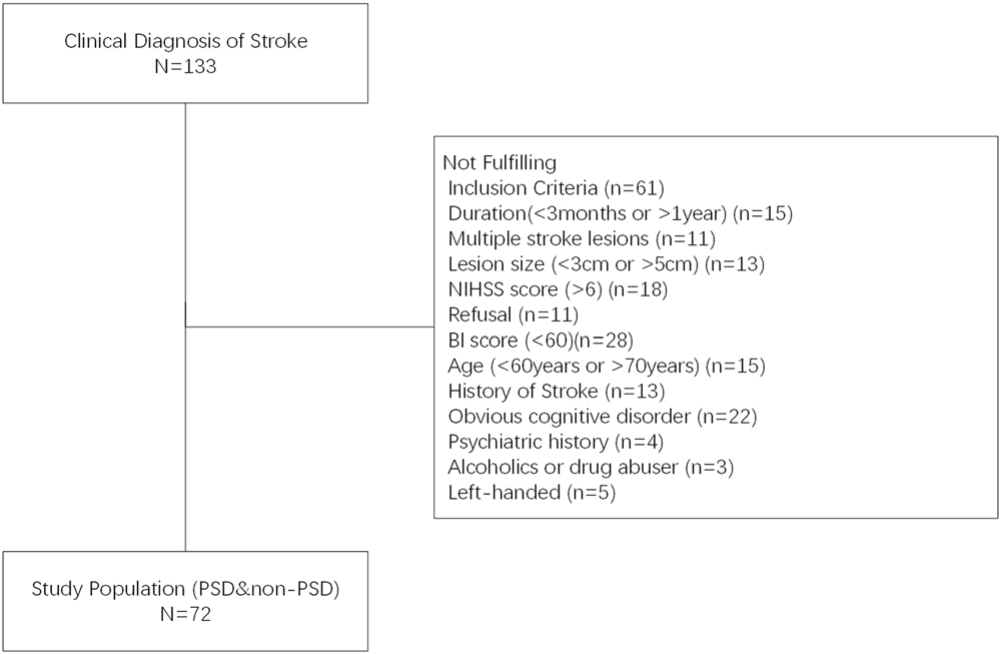
Patients flow in this study. The screened subjects had one or more conditions that did not meet the inclusion criteria.

We reviewed the charts of 133 patients who were admitted for ischemic stroke between December 2012 and June 2017 to the Zhujiang Hospital of Southern Medical University. Patients were included if they met the following criteria. Firstly, the subjects met the WHO criteria for the diagnosis of cerebral infarction, which is based on both the presence of neurological symptoms and a compatible lesion, as demonstrated by magnetic resonance imaging (MRI); secondly, the subjects were in the recovery period (3 months <disease duration <1 year) with stable symptoms; thirdly, the subjects had a single infarcted brain area (3–5 cm) in the right temporal, frontal, or parietal lobe; fourthly, the subjects had a National Institute of Health Stroke Scale (NIHSS) score of six or lower; fifthly, the subjects were conscious and able to cooperate with the interview, and provide informed consent, and complete the scale evaluations and a clinical interview for the diagnosis of depression; sixthly, the subjects had basic self-care ability in their daily lives (Barthel Index score ≥ 60); seventhly; the subjects were aged between 60 and 70 years; eighthly, the subjects did not have a history of hemorrhagic or ischemic stroke; ninthly, the subjects did not have obvious cognitive dysfunction disorder and language understanding disorder; tenthly, the subjects did not have a history of schizophrenia, major depression, anxiety, dementia, drug abuse, or antidepressant use at stroke onset, or a family history of mental disorders; eleventhly, the subjects were not alcoholics or drug abusers. twelfthly, all subjects were right-handed. After a detailed evaluation of inclusion and exclusion criteria, 72 patients who suffered from ischemic stroke participated in this study (Fig. 1).

We also recruited healthy volunteers of similar age, educational background and lifestyle to the stroke patients, with no history of neuropsychiatric disorders or drug abuse. After screening, 15 healthy volunteers were included in the blank control group (baseline).

The following information was collected for each subject: demographics (i.e., age, gender, education level, and whether they lived alone) and stroke severity, as measured by the NIHSS at the time of admission to the hospital. Simultaneously, we obtained scores on the Mini Mental State Examination (MMSE) and the Barthel Index (BI).

All the experiments and protocols were approved by the Ethics Committee of Zhujiang Hospital which is affiliated with the Southern Medical University, China^22^. According to the dictates of the State Council of China, each subject provided written informed consent after receiving detailed instructions and full explanations on the experimental procedures. All methods were performed in accordance with the relevant guidelines and regulations.

The stroke subjects were divided into three subgroups based on their different lesion locations, 1) temporal lobe group, 2) frontal lobe group and 3) parietal lobe group. An experienced neuropsychologist performed the clinical interview to diagnose depression, according to DSM-IV criteria. The severity of depression was assessed using the 24-item Hamilton Rating Scale for Depression (HAMD-24). For inclusion in the depression group (PSD group) in our final analysis, participants had to meet DSM-IV criteria for depressive disorder, and score at least 17 on the HAMD. The other participants were assigned to the non-PSD group.

### Brain imaging

The experiment was performed in the Department of Radiology of Zhujiang Hospital, Southern Medical University, China. Anatomical scans of the brain were collected prior to stimulation imaging. Then, all subjects were subjected to an rs-fMRI scan for six minutes.

Structural and functional scans were acquired with a 3.0 T Philips Achieva MRI System (Royal Philips Electronics, Eindhoven, The Netherlands) with an eight-channel head array coil equipped for echo planar imaging. The images were axial and parallel to the anterior commissure–posterior commissure line, which covered the whole brain. Structural images were collected prior to functional imaging using a T1-weighted fast spin echo sequence (repetition time/echo time = 500/14 ms, flip angle = 90°, 0.859 mm × 0.859 mm in-plane resolution, slice thickness = 1 mm). Blood oxygenation level-dependent functional imaging was acquired using a T2*-weighted, single-shot, gradient-recalled echo planar imaging sequence (repetition time/echo time = 2000/40 ms, flip angle = 90°, 3.4 mm × 3.4 mm in-plane resolution, 180 time points for a total of 360 seconds). In addition, fMRI image collection was preceded by five dummy scans to minimize gradient distortion.

### Preprocessing of experimental functional MRI data

The fMRI image data were preprocessed and analyzed using the Data Processing Assistant for Resting-State fMRI (DPARSF, http://www.restfmri.net) by routines in MATLAB R2010a. The blood oxygen level-dependent (BOLD) time series preprocessing steps included removal of the first 10 volumes, slice-time correction, motion correction, intensity normalization, spatial smoothing, and linear high-pass temporal filtering. The first 10 volumes of each scan were discarded in order to eliminate any non-equilibrium effects of magnetization and to allow subjects to become familiar with the scanning environment. The motion time courses were used to select subjects’ head movements of <2 mm in translation and 2° in rotation (no subjects were excluded). Each individual’s functional images were normalized using the symmetric echo-planar imaging templates and resampled at a resolution of 3 mm × 3 mm × 3 mm. The normalized functional images were smoothed spatially using a 6 mm full width at half maximum (FWHM) Gaussian kernel^23^. Finally, voxel-wise linear trend removal and temporal high-pass filtering (0.01 Hz < f < 0.08 Hz) were applied.

### DC Calculation

Weighted DC measures were calculated using the “REST-DC” toolkit in the REST V1.8 package^18^, as previously described^24^. To obtain each participant’s graph, Pearson correlation coefficients were computed between the time series of all pairs of brain voxels. Each voxel represented a node in the graph, and each significant functional connection (i.e., Pearson correlation) between any pair of voxels was an edge. As a result, we obtained an n × n matrix of Pearson correlation coefficients between any pair of voxels to construct the whole-brain FC matrix for each participant. Then, individual correlation matrices were transformed into a Z-score matrix using Fisher’s r-to-z transformation to improve normality. The weighted DC strength of a voxel as the sum of the connections (Z-values) between a given brain voxel and all other voxels was then computed. To eliminate possible spurious connectivity, we used the Pearson correlation coefficient at r > 0.25 by thresholding each correlation at P ≤ 0.001^18^. Furthermore, standardized weighted DC maps were acquired by subtracting the mean value, and then dividing by the standard deviation within the whole gray matter mask^23^.

### Statistical analysis

SPSS 18.0 software (SPSS, Chicago, IL, USA) was used to calculate descriptive statistics (mean ± SD) for psychophysical data. All statistical assessments were two-tailed, and we considered results to be significant at p < 0.05, consistent with the preliminary status of the trial.

The DC value differences between PSD and non-PSD, and differences between non-PSD and baseline were calculated using two-tailed, paired t-tests (P < 0.05) and corrected for multiple comparisons [false discovery rate [FDR] (P < 0.05) in rest (http://restfmri.net/forum/rest). The resulting images were shown by rest.

## Results

### Patient characteristics

87 subjects (female = 44) were recruited into the study, of whom 72 were stroke patients and 15 were healthy subjects. The mean age of the study sample was 64.60 ± 3.66 (range 60–70) years. Thirty-three of the stroke patients of (45.8%, 33/72) were diagnosed with PSD. There were 21 subjects in the temporal lobe subgroup, 30 subjects in the frontal lobe subgroup, and 21 subjects in the parietal lobe subgroup. A statistical difference was found in the HAMD score between the PSD and non-PSD groups for all three subgroups (p < 0.05). No significant differences were observed in the basic data (i.e., age, sex, education, duration, and whether they lived alone) and functional assessment scores (i.e., MMSE score, BI score and NIHSS score) amongst the three groups (p > 0.05). (See Tables 1 & 2).

**Table 1:** Summary of baseline characteristics of the included subjects.

Group, no. (%) or mean ± SD				
Characteristic	PSD	Non-PSD	Health	p value
n	33	39	15	—
Age, yr	64.26 ± 3.68	64.70 ± 3.95	65.10 ± 2.86	0.46*
Female. Sex	15 (45.5%)	22 (56.4%)	7 (46.7%)	—
Education, yr	8.36 ± 3.24	8.61 ± 3.21	8.11 ± 2.44	0.45*
Duration, mon	7.91 ± 3.00	7.21 ± 2.68	—	0.15^#^
Live alone	6 (18.2%)	5 (12.8%)	5 (33.3%)	—
Lesion size	3.92 ± 0.55	3 0.96 ± 0.66	—	0.39^#^
HAMD score	20.63 ± 2.41	4.93 ± 1.56	—	<0.05^#^
MMSE score	23.36 ± 2.09	24.16 ± 1.99	—	0.06^#^
BI score	73.73 ± 14.98	74.78 ± 18.78	—	0.39^#^
NIHSS score	2.2 ± 1.4	1.6 ± 1.7	—	0.06^#^

BI = Barthel Index; MMSE = Mini Mental State Examination; NIHSS = National Institutes of Health Stroke Score; SD = standard deviation. *ANOVA test, ^#^T test.

**Table 2:** Summary of baseline characteristics of the 72 post-stroke patients.

Group, no. (%) or mean ± SD			
Characteristic	PSD	Non-PSD	p value
Temporal lobe subgroup			
n	9	12	—
Age, yr	65.22 ± 3.89	63.16 ± 4.88	0.15
Female. Sex	5 (55.6%)	7 (58.3%)	0.90
Education, yr	9.28 ± 2.56	8.51 ± 3.87	0.31
Duration, month	8.98 ± 2.45	7.69 ± 3.25	0.17
Live alone	2 (22.2%)	1 (8.3%)	0.16
Lesion size	3.81 ± 0.67	3.89 ± 0.71	0.40
HAMD score	19.38 ± 1.24	4.56 ± 1.39	<0.05*
MMSE score	23.52 ± 1.64	24.28 ± 2.24	0.20
BI score	74.18 ± 13.87	72.15 ± 17.58	0.40
NIHSS score	2.0 ± 1.9	1.5 ± 1.7	0.27
Frontal lobe subgroup			
n	13	17	—
Age, yr	64.39 ± 4.01	65.88 ± 4.23	0.17
Female. Sex	4 (30.8%)	10 (58.8%)	0.16
Education, yr	7.69 ± 3.44	9.24 ± 2.98	0.10
Duration, month	6.98 ± 3.80	7.14 ± 2.15	0.44
Live alone	3 (23.1%)	2 (11.8%)	0.23
Lesion size	3.98 ± 0.55	4.05 ± 0.63	0.38
HAMD score	20.18 ± 1.38	5.22 ± 1.92	<0.05*
MMSE score	22.20 ± 2.37	23.45 ± 1.94	0.06
BI score	72.76 ± 17.11	76.14 ± 20.16	0.31
NIHSS score	2.6 ± 1.2	1.8 ± 1.9	0.10
Parietal lobe subgroup			
n	11	10	—
Age, yr	66.27 ± 3.17	64.56 ± 4.35	0.21
Female. Sex	6 (54.5%)	5 (50%)	0.59
Education, yr	8.39 ± 3.56	7.64 ± 2.77	0.30
Duration, month	8.12 ± 2.18	6.74 ± 2.95	0.11
Live alone	1 (9.1%)	2 (20.0%)	0.38
Lesion size	3.95 ± 0.49	3.91 ± 0.68	0.44
HAMD score	22.13 ± 3.25	4.88 ± 1.02	<0.05*
MMSE score	24.55 ± 1.35	25.24 ± 1.43	0.13
BI score	74.49 ± 14.58	75.67 ± 19.38	0.37
NIHSS score	1.9 ± 1.3	1.2 ± 1.4	0.12

BI = Barthel Index; MMSE = Mini Mental State Examination; NIHSS = National Institutes of Health Stroke Score; SD = standard deviation.

### Hemodynamic responses

**Figure 2:**
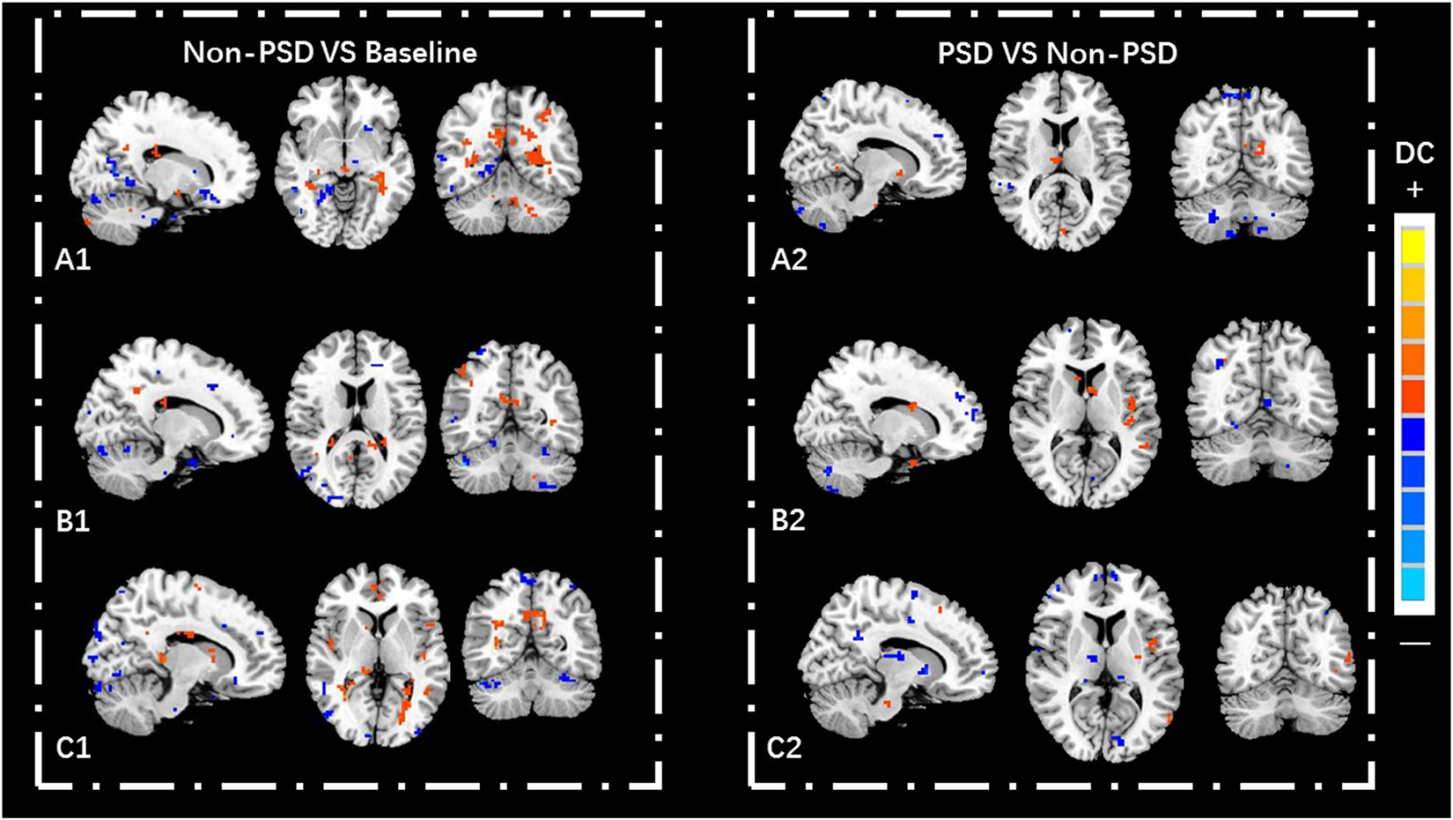
Differences in DC values between non-PSD patients and the baseline, and between PSD and non-PSD patients in the three subgroups, (**A**): temporal lobe subgroup, (**B**): frontal lobe subgroup, (**C**): parietal lobe subgroup.

Compared with the baseline (healthy volunteers), the DC values of three non-PSD subgroups varied in several brain areas such as prefrontal cortex, limbic lobe, motor cortex, sensory cortex, temporal cortex, parietal cortex and cingulate cortex (See Table 3; Fig. 2).

**Table 3:** The DC values of non-PSD in three subgroups (P < 0.05, FDR < 0.05).

MNI							
Brain region	BA	R/L	X	Y	Z	Voxel	DC value
Temporal lobe subgroup							
Cerebellum Anterior Lobe		R	48	−45	−33	460	−25.1552
Cerebellum Posterior Lobe		L	−21	−48	−45	162	13.4981
Brainstem		L	−15	−42	−42	145	12.4268
OFC	47	R	15	30	−18	167	−6.4142
OFC	11	L	−6	33	−18	98	9.5739
DLPFC	47	L	−42	21	−15	115	−13.0923
Superior Temporal Gyrus	38	L	−54	15	−15	267	−18.039
Superior Temporal Gyrus	41	R	51	−24	9	145	−13.0716
PHP	20	L	−27	−9	−39	133	9.0581
PHP	19	R	18	−60	−18	167	−10.593
Amygdala	28	L	−24	−3	−9	247	16.8704
HP	19	R	18	−45	−3	136	−18.3167
ACC		R	1	24	21	65	−11.0309
PCC		R	6	−51	27	327	24.5249
Thalamus		R	3	−21	15	85	−8.5049
Insular	22	L	−48	−15	9	287	24.4996
Angular Gyrus	39	L	−48	−69	27	196	23.9556
Lingual Gyrus	18	L	−18	−84	−18	117	−12.9231
Postcentral Gyrus		R	51	−15	12	87	10.0361
Precentral Gyrus	4	L	−18	−27	66	115	13.3974
Frontal lobe subgroup							
Cerebellum Posterior Lobe		R	42	−57	−36	347	−79.5966
Cerebellum Posterior Lobe		L	−27	−36	−48	89	−7.8308
Brainstem		R	9	−18	−33	167	−10.07
OFC	11	L	−30	24	−21	154	−11.7089
VMPFC		R	12	39	−6	173	−9.0333
DLPFC	47	L	−39	21	−3	128	−9.2105
DLPFC		R	30	42	21	89	−5.1952
Middle Temporal Gyrus	21	R	57	−24	−18	102	−11.0803
PHP		R	18	0	−30	147	−12.9329
PHP		L	−30	0	−18	77	6.6728
Amygdala	28	L	−27	−18	−21	245	21.4537
Insular	13	R	36	12	−6	198	−15.1015
Insular		L	−42	−24	21	139	13.6333
ACC		L	−15	36	15	79	−11.7163
ACC	24	R	6	21	24	140	−16.6639
PCC	31	L	−6	−39	36	263	14.5737
Angular Gyrus	40	R	60	−54	27	89	7.5325
Inferior Parietal Lobule	40	R	51	−30	24	67	−9.0799
Precentral Gyrus	4	L	−42	−15	51	45	−10.0119
Postcentral Gyrus		R	3	−51	69	89	−18.2157
SMA		R	6	15	42	87	−7.1809
Parietal lobe subgroup							
Cerebellum Anterior Lobe		R	42	−48	−36	267	−31.9494
Cerebellum Posterior Lobe		L	−42	−75	−48	56	20.75
Brainstem		L	−21	−18	−6	90	−5.3091
OFC	11	R	6	48	−21	117	11.2343
VMPFC	10	L	−3	57	9	156	24.8301
DLPFC	10	R	18	45	15	89	−7.4365
Middle Temporal Gyrus	21	L	−51	−30	−3	118	12.7905
Amygdala		L	−24	0	−15	81	5.8148
PHP		L	−21	−39	−6	127	11.6859
ACC	32	R	6	21	−9	337	−41.5189
PCC		L	−9	−51	9	67	5.5755
PCC	30	R	3	−48	18	85	7.21
Insula	13	L	−45	−18	0	129	13.3885
Thalamus		R	12	−33	3	248	12.9741
Angular Gyrus	40	L	−60	−51	24	87	9.9668
Superior Parietal Lobule	7	L	−33	−66	57	118	−5.8638
Cuneus		R	15	−69	18	67	−14.6015
Precuneus	19	L	−24	−84	36	115	−18.428
Precuneus		R	2	−57	66	89	−14.5688
Postcentral Gyrus		R	42	−27	42	77	−6.8154
Postcentral Gyrus		L	−45	−21	48	189	−40.6064
Precentral Gyrus	4	R	33	−24	72	134	−12.0003
SMA	6	L	−9	9	72	84	5.7901

Abbreviations: FDR, false discovery rate; MNI, Montreal Neurologi-cal Institute. The negative values of Peak (−) represent the decrease degree centrality value, and the positive values of Peak (+) represent the increase degree centrality value. PHP: parahippocampal gyrus; HP: hippocampal gyrus; VMPFC: ventromedial prefrontal cortex; DLPFC: dorsolateral prefrontal cortex; ACC: anterior cingulate cortex; MCC: mid-cingulate cortex; SMA: supplementary motor area.

**Figure 3:**
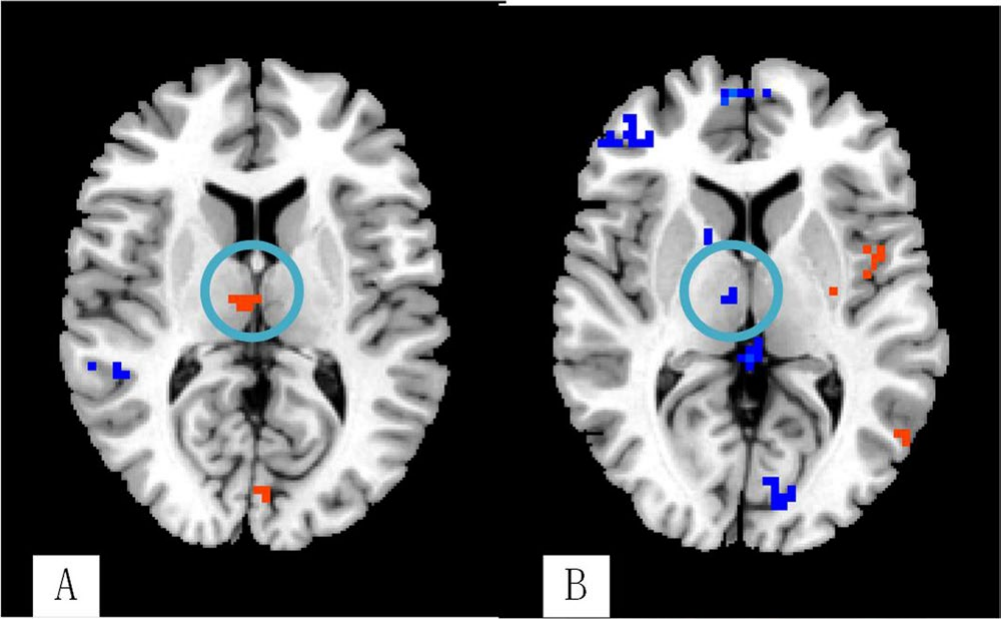
Right thalamus showing different status in different subgroups; (**A**): temporal lobe subgroup, (**B**): parietal lobe subgroup.

**Figure 4:**
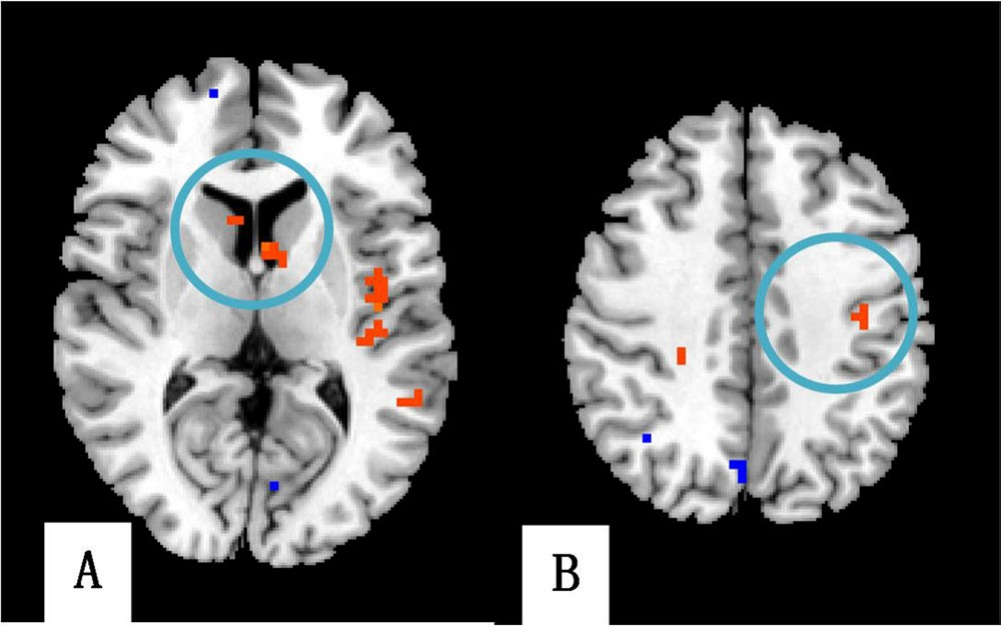
DC values of frontal lobe subgroup showing specific brain areas; (**A**) caudate, (**B**) postcentral gyrus.

**Figure 5:**
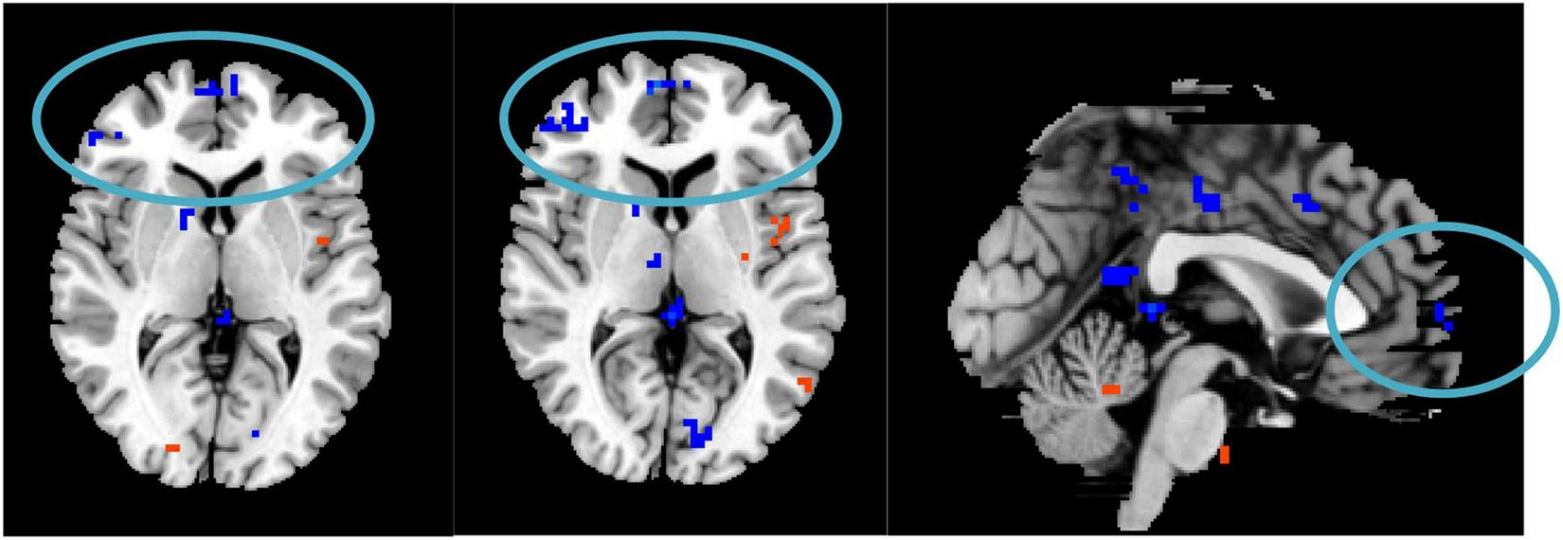
DC values of frontal lobe brain areas in the parietal lobe subgroup.

Compared with the non-PSD group, several brain regions of PSD showed decreased DC values, e.g., the ventromedial prefrontal cortex (VMPFC), dorsolateral prefrontal cortex (DLPFC), hippocampal gyrus (HP), thalamus, posterior cingulate cortex (PCC), angular gyrus, and cerebellum lobe. However, DC values were greater in brain areas such as the temporal gyrus, parahippocampal gyrus (PHP), insula, anterior cingulate cortex (ACC), caudate, and supplementary motor area (SMA) (See Table 4; Fig. 2). The brain network of each subgroup showed distinct characteristics. For example, our research demonstrated that the right thalamus in the temporal lobe subgroup showed higher DC values than the other two subgroups (See Table 4 & Fig. 3). In the frontal lobe subgroup, the DC values of the left DLPFC, caudate, and postcentral gyrus were higher than the other two subgroups (See Table 4 & Fig. 4). In addition, for the parietal lobe subgroup, the frontal lobe area of the brain showed decreased DC values compared to the other subgroups (See Table 4 & Fig. 5).

**Table 4:** The DC values of PSD in three subgroups (P < 0.05, FDR < 0.05).

MNI							
Brain region	BA	R/L	X	Y	Z	Voxel	DC value
Temporal lobe subgroup							
Cerebellum Posterior Lobe		R	3	−60	−57	131	−10.4592
Cerebellum Posterior Lobe		L	−24	−72	−48	672	−10.5024
Brainstem		R	3	−15	−30	316	17.4211
Superior Temporal Gyrus	38	R	45	12	−33	174	11.2886
Middle Temporal Gyrus		L	−48	−21	−18	156	6.458
PHP	34	L	−9	3	−18	479	7.5107
PHP	30	R	18	−54	−3	156	7.0346
VMPFC	11	L	−24	45	−15	183	−15.8736
VMPFC	9	R	18	51	24	253	−15.4473
DLPFC	47	L	−48	33	0	111	−21.126
DLPFC	10	R	30	48	27	179	−12.4312
Insula	13	R	39	−15	−9	234	8.0511
Insula	13	L	−39	−18	−6	373	18.9759
ACC	11	R	6	42	−9	280	11.8538
MCC	24	L	−3	−9	39	245	−8.641
Thalamus		R	3	−12	15	67	8.4677
Lateral Globus Pallidus		R	6	−3	−9	190	6.0041
Superior Parietal Lobule	7	L	−24	−54	48	117	−11.3082
Inferior Parietal Lobule	40	R	57	−45	45	348	8.0849
Postcentral Gyrus	6	L	−3	−36	75	782	−25.1996
frontal lobe subgroup							
Cerebellum Posterior Lobe		R	18	−75	−51	281	−12.6249
Brainstem		L	−3	−15	−39	496	13.0872
Superior Temporal Gyrus	38	L	−45	12	−21	142	7.2025
Middle Temporal Gyrus	20	L	−45	−3	−21	387	11.9711
PHP	28	R	15	6	−33	161	9.693
HP		L	−33	−12	−21	263	−7.0883
DLPFC	38	L	−45	15	−12	195	12.8502
DLPFC	46	R	54	39	3	111	−7.6109
VMPFC	10	R	15	60	18	534	−9.0539
Insula	6	L	−42	−15	9	173	13.7238
ACC		R	15	6	21	222	11.467
ACC		L	−6	12	24	379	11.6119
Thalamus		L	−12	−21	3	111	−27.3421
Caudate		L	−3	6	9	134	13.0255
Caudate		R	3	9	12	125	11.7595
Lingual Gyrus	30	L	−3	−84	3	379	−15.0398
Angular Gyrus	39	R	36	−60	45	253	−13.4331
Inferior Occipital Gyrus	19	L	−30	−81	−21	168	−15.867
Superior Occipital Gyrus	19	L	−39	−87	27	621	−6.3595
Postcentral Gyrus	2	R	42	−24	39	415	11.0299
Postcentral Gyrus	4	L	−39	−15	42	114	6.8746
parietal lobe subgroup							
Cerebellum Posterior Lobe		L	−30	−84	−27	111	−6.4658
Cerebellum Anterior Lobe		R	9	−27	−33	468	26.9807
Brainstem		L	−12	−12	−45	139	28.4617
Superior Temporal Gyrus	21	L	−51	3	−3	487	13.9493
Middle Temporal Gyrus	19	L	−57	−66	12	116	46.8026
PHP	36	L	−27	−30	−27	243	9.2998
HP		R	36	−12	−24	118	−10.3216
Inferior Frontal Gyrus	11	R	48	48	−12	321	−7.3132
Middle Frontal Gyrus	10	R	42	51	9	127	−18.0391
Superior Frontal Gyrus	6	L	−21	−3	63	179	−14.0965
VMPFC	10	R	6	60	6	256	−21.1974
VMPFC	10	L	−9	60	12	157	−21.3496
Insula	44	L	−42	3	9	193	8.5406
Insula		L	−33	−12	12	212	12.2319
Thalamus		R	12	−12	9	398	−10.4573
Thalamus		L	−15	−24	18	266	−25.933
PCC	23	L	−3	−42	21	154	−9.6221
PCC	31	R	12	−51	27	237	−11.3722
MCC	24	R	6	−12	36	289	−9.1077
MCC	31	L	−6	−21	39	274	−9.055
Angular Gyrus		L	−30	−51	39	115	−8.2101
Middle Occipital Gyrus	18	L	−42	−93	−3	137	31.8151
Lentiform nucleus		R	12	9	0	237	−15.3808
Postcentral Gyrus	3	R	36	−21	45	149	−10.2303
SMA	8	R	9	21	54	116	11.7321

Abbreviations: FDR, false discovery rate; MNI, Montreal Neurologi-cal Institute. The negative values of Peak (−) represent the decrease degree centrality value, and the positive values of Peak (+) represent the increase degree centrality value. PHP: parahippocampal gyrus; HP: hippocampal gyrus; VMPFC: ventromedial prefrontal cortex; DLPFC: dorsolateral prefrontal cortex; ACC: anterior cingulate cortex; MCC: mid-cingulate cortex; SMA: supplementary motor area.

## Discussion

Post-stroke depression (PSD) is a common complication of stroke that has a negative impact on the rehabilitation process, and always brings a great economic burden for patients^25^. clarification of the mechanism of PSD is very important, and may be achieved through fMRI, which provides an effective tool for the study of the brain network of the condition.

In the present study, no statistical differences were observed in the basic data (i.e., age and gender) amongst the three groups. This result suggests that, under the strict inclusion criteria, differences in age and gender amongst the three subgroups had minor influence. This led to reduced heterogeneity of the experiment and improvements in the reliability of the conclusion. Previous studies have shown that PSD results from brain injury and nerve damage caused by stroke, the severity of which is an important factor in PSD^26^. In our study, there were no significant differences in NIHSS score, MMSE score and BI score between the PSD and non-PSD groups. Although the stroke subjects showed similar degrees of nerve damage, some individuals did not appear to present depressive symptoms. This suggests that nerve damage may not be a key determining factor for PSD, and other factors are more influential in the onset of depression.

### Non-PSD brain response

Our results indicate that non-PSD can extensively alter the excitability of the brain network, including stroke lesions and remote sites. These altered brain networks are closely associated with emotion, cognition, memory, sensation, movement, etc. We believe that strokes can affect these networks and cause emotional, sensory, motor and other dysfunctions in patients. In addition, the brain responses of different subgroups were not identical and showed different characteristics. For instance, wide inactivation in the limbic system (i.e., insular, ACC, thalamus, and PHP) in the frontal lobe subgroup was apparent, whilst the parietal lobe subgroup showed limbic system over-activation. The main reason for this difference may be that the parietal lobe is predominantly involved in sensory, taste and logical functions^27^, and is not closely related to the limbic system. In contrast, the prefrontal cortex is closely related to the limbic system, with numerous axons connecting each other. Prefrontal cortex and limbic systems are involved in higher brain functions such as emotion and cognition^28^. Frontal stroke causes disruption of nerve connections and decreased transmission of information, resulting in decreased activity of the limbic system. Different brain network reactions can cause different prognosis of stroke patients, the nature of which deserves further attention.

### Similar emotional network of PSD

Using structural and functional MRI data from the subjects, we showed that brain areas located in the prefrontal lobe, thalamus, PCC, angular gyrus, and cerebellum had decreased DC values in the three subgroups. Additionally, the temporal lobe, PHP, insular, ACC, and SMA had increased DC values in these subgroups. These results suggest that strokes of different lesion locations have similar brain networks.

#### Negative brain network

The DLPFC, thalamus, HP, and PCC belong to the limbic system. The limbic system supports a variety of functions including emotion, behavior, motivation and memory^29^. Emotional life is largely housed in the limbic system which is the site of generation and conduction of emotional information^30^. The decreased DC values of the limbic system may affect emotional information processing, which plays a role in PSD. As the functional connection of the limbic system reduces, the whole network is left in a restrained state, which affects other functions of the brain network.

The prefrontal lobe is well known to have a wide range of neural connections and complex structural schemas, as well as rich and complex axonal linkages, which are involved in the processing of emotional information^31^. Decreased DC values of the prefrontal lobe would lead to a reduction in the neural association between the prefrontal lobe and other brain regions, thereby reducing the transmission of emotional information. In addition, the left prefrontal cortex is closely related to the reward system, which can promote the secretion of dopamine^32^. The decreased DC values of the left prefrontal cortex may reduce the frequency of the reward mechanism function, and aggravate the negative state of stroke patients.

The thalamus is an important part of the limbic system, which is associated with changes in emotional reactivity^33^. In emotional conduction, the thalamus shares a nerve connection with multiple brain areas. For example, the medial dorsal nucleus makes connections with cortical zones of the prefrontal lobe^34^. Also, the anterior nuclei connect with the mammillary bodies, and through them (via fornix), with the HP and the CC^35^. Based on these phenomena, some researchers believe that the thalamus is the relay station of emotional conduction. In our study, the thalamus showed a decreased DC value, which would result in reduced nerve conduction among the prefrontal lobe, HP, CC and thalamus. Moreover, some studies have shown that the frontal lobe and thalamus together constitute the awareness system, which is the main center of spiritual activity (32). Decreased functional connectivity in both brain regions may affect the awareness system, and is a possible reason for the slow-thinking symptom of PSD.

Most researchers believe that the HP is associated with recent memory, and involved in emotional reactions and control^36^. Decreased DC values of the HP would affect these emotional functions, and may be the reason that PSD patients are restless and easily angered. Likewise, the decreased memory function of HP also causes memory impairment in PSD patients. The PCC has been strongly linked to emotional salience. Our study showed that the PCC of both sides had decreased DC values; PCC abnormalities may be associated with the negative memory of depressed patients, which is thought to be related to emotional burden.

The prefrontal lobe, PCC, HP and angular gyrus belong to the default mode network (DMN). The DMN has two apparently opposite functions: a spontaneous cognition function and the function of monitoring the environment (sentinel hypothesis)^37^. Our results suggest that PSD may significantly inhibit the functional activities of the DMN, and thereby weaken both of these functions. In addition, PSD may reduce self-evaluation and alerts to environmental awareness, eventually leading to lower episodic memory and alertness, all of which play an important role in the PSD network.

#### Positive brain network

In the present study, some brain areas including the temporal lobe, PHP, insular, and ACC showed increased DC values, in contrast to previous experiments^38^. The insular is believed to be involved in consciousness, and plays a role in diverse functions usually linked to emotion^39^. The anterior insular cortex is thought to be responsible for emotional feelings, and processes a person’s sense of disgust in society. The increased DC values of the insular may aggravate the disgust symptoms of PSD patients. Moreover, as the insular and ACC are important nodes of the pain network^40^, their excessive activation may also explain why many PSD patients suffer pain in the recovery period.

The ACC is involved in higher-level functions such as decision-making, impulse control, and emotion^41^. Excessive activation of the ACC has been linked to the transmission of negative emotions. Other studies suggest that the temporal lobe controls the emotion response; if the temporal lobe appears negative during activation, the mood of the patient will stabilize^42^. In our study, the temporal lobe showed increased DC values, which may explain the irritability observed in PSD patients.

Briefly, the similar emotional network consists of a positive and a negative network. The positive network is associated with the generation of the emotion of disgust, negative emotional transmission, and stimulation of a pain sensation, and includes the temporal lobe, PHP, insular, ACC, etc. The negative network is mainly linked to decreased functional connection, emotional network and alertness inhibition, the generation of negative emotions, and awareness system inhibition, and includes brain areas in the limbic system and the DMN. The similar emotional network may be the common neurophysiological basis of PSD.

### Individual emotional network of PSD

Our experimental results indicate that each subgroup has its own characteristics. In the temporal lobe subgroup, the right thalamus had increased DC values. The temporal lobe is involved in emotional processes, and is responsible for recognizing familiar facial emotions and interpreting emotions through someone’s body posture. Evidence suggests that the temporal lobe may also be involved in precipitating emotional empathy^43^. Damage to this area can result in problems with memory, understanding language, and maintaining emotional control. Extensive damage to the temporal lobe caused by stroke leads to emotional control reduction of the ipsilateral cerebral hemisphere^42^, and an increase in unstable negative nerve impulses. The thalamus is the relay station of emotional conduction, and is directly connected with the temporal lobe. Stroke injury causes the ipsilateral thalamus to transmit more negative emotions, leading to enhanced thalamus activation.

In the frontal lobe subgroup, the left DLPFC and caudate showed increased DC values which were different from the other subgroups. Based on the mechanism of plasticity^44^, the brain will form compensatory areas in the contralateral hemisphere. As a consequence of damage to ipsilateral brain function, DLPFC (as an important node of the limbic system and DMN) needs to transmit a high level of negative emotional information. Thus, more neurons are required for transmission in contralateral compensatory brain areas, which eventually leads to enhanced compensatory brain area activation. In addition, the compensatory activation of the DLPFC may cause activation of dopamine-related brain areas such as caudate, probably due to the close association between LPFC and the dopamine secretion mechanism^45^.

In the parietal lobe subgroup, the frontal lobe showed substantially larger areas of decreased DC values than the other two subgroups. The parietal lobe integrates sensory information among various modalities, including spatial sense and navigation, and is responsible for brain information integration^46^. Extensive activation of the prefrontal cortex may be due to the confusion within the integrated system of the damaged parietal lobe.

In brief, each subgroup possessed its own characteristics in terms of lesion locations. Four factors contributive to this observation: the neurological function of the stroke site, the compensatory mechanism of the brain, the mechanism of integrity and the locality of the brain.

### Study limitations

Although the subjects were divided into three subgroups and heterogeneity reduced, more lesion locations need to be assessed to improve the reliability of the conclusions. Furthermore, the brain network of PSD of different generations, durations, and lesion sizes require analysis. The sample size in this study was relatively small, and future experiments should recruit more participants to achieve robust conclusions. Moreover, DC measurments require a threshold (r > 0.25), which represents a shortcoming of our experimental method. Thus, we plan to use more advanced methodology in future studies, including participation coefficient and eigenvector centrality. Furthermore, multimodal brain imaging (i.e., diffusion tensor imaging and voxel-based morphometry) is useful for studying gray matter density and anatomical connectivity of the PSD brain, and will be employed in future experiments.

## Conclusion

The hemodynamic response of PSD indicates that PSD has activities of similar emotional networks, of which the negative network realizes its function through the limbic system and DMN. The brain network has unique characteristics for different lesion locations. The neurological function of the lesion location, the compensatory mechanism of the brain, and the mechanism of integrity and locality of the brain are the important factors in the individual emotional network.
